# Association between blood pressure and circadian timing of physical activity of Japanese workers

**DOI:** 10.3389/fphys.2022.992945

**Published:** 2022-09-26

**Authors:** Momoko Imamura, Yu Tahara, Takahiko Suiko, Yuki Nagamori, Shigenobu Shibata

**Affiliations:** ^1^ Laboratory of Physiology and Pharmacology, School of Advanced Science and Engineering, Waseda University, Tokyo, Japan; ^2^ Graduate School of Biomedical and Health Sciences, Hiroshima University, Hiroshima, Japan; ^3^ Research and Development Headquarters, Lion Corporation, Tokyo, Japan

**Keywords:** blood pressure, circadian clock, worker, exercise, hypertension

## Abstract

High blood pressure (BP) is reported to be accounted for more than 10 million deaths, and the high prevalence of hypertension is a global issue. Exercise is known to reduce BP and the optimal exercise prescription has been discussed. Furthermore, since the circadian clock plays an important role in BP regulation and its related physiological functions, the time-of-day difference in the effect of exercise on BP is suggested. However, when people should regularly exercise for the prevention of hypertension remains unclear. In this cross-sectional research, we analyzed the association of habitual exercise and BP depending on their performed timing, time length, and frequency for three levels of intensity from an analysis of questionnaire answered by Japanese male workers (*N* = 2,343, mean age ± SE = 49.2 ± 0.2 years old, date: June 2021). From the subjects who responded, subjects with irregularly high or low BP, no regular physical activity or under the treatment of hypertension were excluded from the analysis. From the comparison of SBP and DBP between those who performed physical activity at each time period, vigorous or moderate physical activity in the evening (18:00–21:00) showed significantly lower average BP. On the other hand, those who walked in early morning (03:00–06:00) showed higher DBP. These time-specific differences were confirmed from multiple regression analysis, eliminating the confounding factors such as age and BMI. When participants were divided into groups according to their BP, compared to normal BP groups, higher BP groups exercised less in the evening. Our findings suggest that when to exercise is important, and that exercising in the evening (18:00–21:00) may be better to achieve the hypotensive effect of exercise.

## 1 Introduction

Hypertension is highly prevalent globally, and 1.28 billion people aged between 30–79 years old are estimated to have this disease (WHO, 2021). Furthermore, high systolic blood pressure (SBP) was reported to be accounted for 10.8 million deaths worldwide (Collaborators, 2020). Thus, strategies to manage high blood pressure (BP) are essential.

Exercise is known to reduce BP, and this is strongly supported by a large number of studies (Diaz and Shimbo, 2013). In a longitudinal study, an inverse association was confirmed between an increasing pattern of cardiorespiratory fitness and incident hypertension (Sui et al., 2017). Exercise prescription is characterized by the Frequency, Intensity, Time, and Type (FITT) principle. The optimal FITT for the prevention of hypertension has been discussed. Because of the acute BP lowering after exercise, known as post-exercise hypotension (PEH), exercising most of the week is recommended (Pescatello et al., 2015). Moderate intensity physical activity has been recommended in many groups. While Pavey et al. reported that vigorous physical activity does not provide additional benefits above those from the moderate physical activity (Pavey et al., 2013), some studies reported that more rigorous intensity leads to greater BP reductions (Pescatello et al., 2015). For time, continuous or accumulated in the day, a total of 150 or more minutes per week has been agreed to be favorable (Thompson et al., 2013). Aerobic exercise is the type of exercise which has been highly recommended, and many interventions and meta-analysis have confirmed the BP lowering effect. Although the evidence is yet weak, resistance training has also shown a hypotensive effect (Cornelissen and Smart, 2013). Another meta-analysis revealed that walking alone can lower BP as well, regardless of age, sex, or baseline BP (Lee et al., 2021).

The mechanism of the hypotensive effect of exercise remains unclear, but animal studies have reported the association of insulin sensitivity, autonomic nervous system function, vasoconstriction regulation, and more factors (Araujo et al., 2013; Moraes-Silva et al., 2013). The circadian clock plays an important role in these physiological functions (Bass and Takahashi, 2010). Moreover, BP exhibits a circadian rhythm, with a sharp rise in the morning and dipping at night (Smolensky et al., 2017). In our recent study, we have shown that meal-specific associations with BP exist in certain nutrients depending on intake timing (Imamura et al., 2022). For example, the sodium/potassium ratio of food is negatively associated with blood pressure at lunch time but not at dinner time (Imamura et al., 2022). Thus, focusing on timing may be important for BP. Furthermore, other time-dependent therapeutic effects of exercise have been reported. Acute response of fat oxidation is greater in the evening while the effects of exercise are not observed before breakfast (Aoyama and Shibata, 2020). In mice, the increase in post-prandial physical activity rather than pre-prandial physical activity increased short-chain fatty acids and altered the microbiota composition (Sasaki et al., 2022). Therefore, exercise may also show different effects on BP depending on the timing of the day. Previous studies have compared the acute effects of aerobic exercise performed in the morning and evening, drawing different conclusions. Jones et al. reported that only evening exercise showed hypotensive effects (Jones et al., 2008). Taking the circadian rhythm into account, a larger decrease in SBP was observed with morning exercise (de Brito et al., 2015). In a 10-weeks intervention among treated hypertensive men, Brito et al. reported that only evening exercise decreased SBP (Brito et al., 2019). The timing difference of exercise on BP is suggested, yet when people should regularly exercise for the prevention of hypertension remains unclear.

In this research, we aim to discover the association between habitual exercise and BP depending on their timing in addition to the time length, intensity, and frequency from an analysis of questionnaire answered by Japanese male workers.

## 2 Materials and methods

### 2.1 Questionnaire

In this cross-sectional study, an online survey was conducted on Japanese male workers aged 20–49 years old. Basic characteristics (age, sex, and BMI), the subjects’ physical activity habits, and blood pressure were obtained from the questionnaire.

This survey was conducted from June 18 until 22 June 2021. This experiment has been approved by the ethics review committee on research with human subjects in Waseda University and Lion Corporation (No. 2020–046 and No. 352, respectively), and followed the guidelines laid down in the Declaration of Helsinki.

### 2.2 Physical activity

The subjects were asked about their physical activity habits of the past 4 weeks. For different levels of physical activity intensity, from vigorous physical activity, moderate physical activity, and walking, subjects were asked how many times they exercise a week, and for how long they exercise on the day they exercise, and at what time period they usually exercise. The time period was answered from a choice of 00:00 to 03:00 (00–03), 03:00 to 06:00 (03–06), 06:00 to 09:00 (06–09), 09:00 to 12:00 (09–12), 12:00 to 15:00 p.m. (12–15), 15:00 to 18:00p.m. (15–18), 18:00 to 21:00 (18–21), 21:00 to 24:00 (21–24), and Irregular timing (Irregular). We calculated weekly metabolic equivalents (MET) based on the International Physical Activity Questionnaire (IPAQ) analysis guidelines for each level of physical activity intensity (Group, 2005). The IPAQ is widely used to assess physical activity (Craig et al., 2003).

Each METs were calculated as shown below.

Vigorous MET-minutes/week = 8.0 * vigorous-intensity activity minutes/day (Time length) * vigorous-intensity activity days/week (Frequency).

Moderate MET-minutes/week = 4.0 * moderate-intensity activity minutes/day (Time length) * moderate activity days/week (Frequency).

Walking MET-minutes/week = 3.3 * walking minutes/day (Time length) * walking activity days/week (Frequency).

Total physical activity MET-minutes/week = sum of Walking + Moderate + Vigorous MET- minutes/week scores.

Vigorous physical activity was defined as any activity that feels physically demanding and makes the subject out of breath (e.g., carrying heavy loads, biking up hills, jogging, tennis singles, etc.). Moderate physical activity was defined as any activity that feels somewhat physically demanding and makes the subject a little breathless (e.g., carrying light loads, playing tag with children, slow swimming, tennis doubles, golf without a cart, etc.). Walking was defined as any kind of continuous walking for more than 10 minutes in work or daily life. Data cleaning, such as excluding of outliers was performed following the guidelines established by the IPAQ (Group, 2005).

### 2.3 Blood pressure

SBP [mmHg] and diastolic blood pressure (DBP [mmHg]), measured at a hospital or during a medical checkup/examination were answered by the subjects. In Japan, workers annually take a medical checkup, and this data is known to be reliable. For the later analysis, subjects were grouped according to their SBP and DBP based on the Japanese blood pressure classification guideline (Umemura et al., 2019). Subjects were divided into Normal BP (SBP<120 and DBP<80), High normal BP (120≦SBP≦129 and DBP<80), Elevated BP (130≦SBP≦139 and/or 80≦DBP≦89), Hypertension (SBP≧140 and/or DBP≧90), and (Isolated) Systolic hypertension (SBP≧140 and DBP<90) groups.

### 2.4 Statistical analysis

The obtained data was statistically analyzed using a predictive analytics software for Windows (Statistical Package for the Social Sciences; IBM Corp., Chicago, IL, United States). The sample size was decided using G*Power prior to the study (G*Power, version 3.1.9.2, Universitat Kiel, Germany). To examine the effect of physical activity on BP, physical activity levels were compared among categorized BP groups using a one-way analysis of variance (ANOVA) with Tukey post-hoc test. To investigate the relationship between physical activity timing and blood pressure, the mean BP of those who performed physical activity and those who did not were compared using student T-test. To clarify the relationship and the interactions between time periods excluding other factors, multiple regression analysis was conducted. Comparison of the physical activity among the categorized BP groups were conducted using a one-way ANOVA with Tukey post-hoc test. A *p*-value of <0.05 was considered statistically significant. Data are expressed by mean and standard error.

## 3 Results

### 3.1 Subject characteristics

**FIGURE 1:**
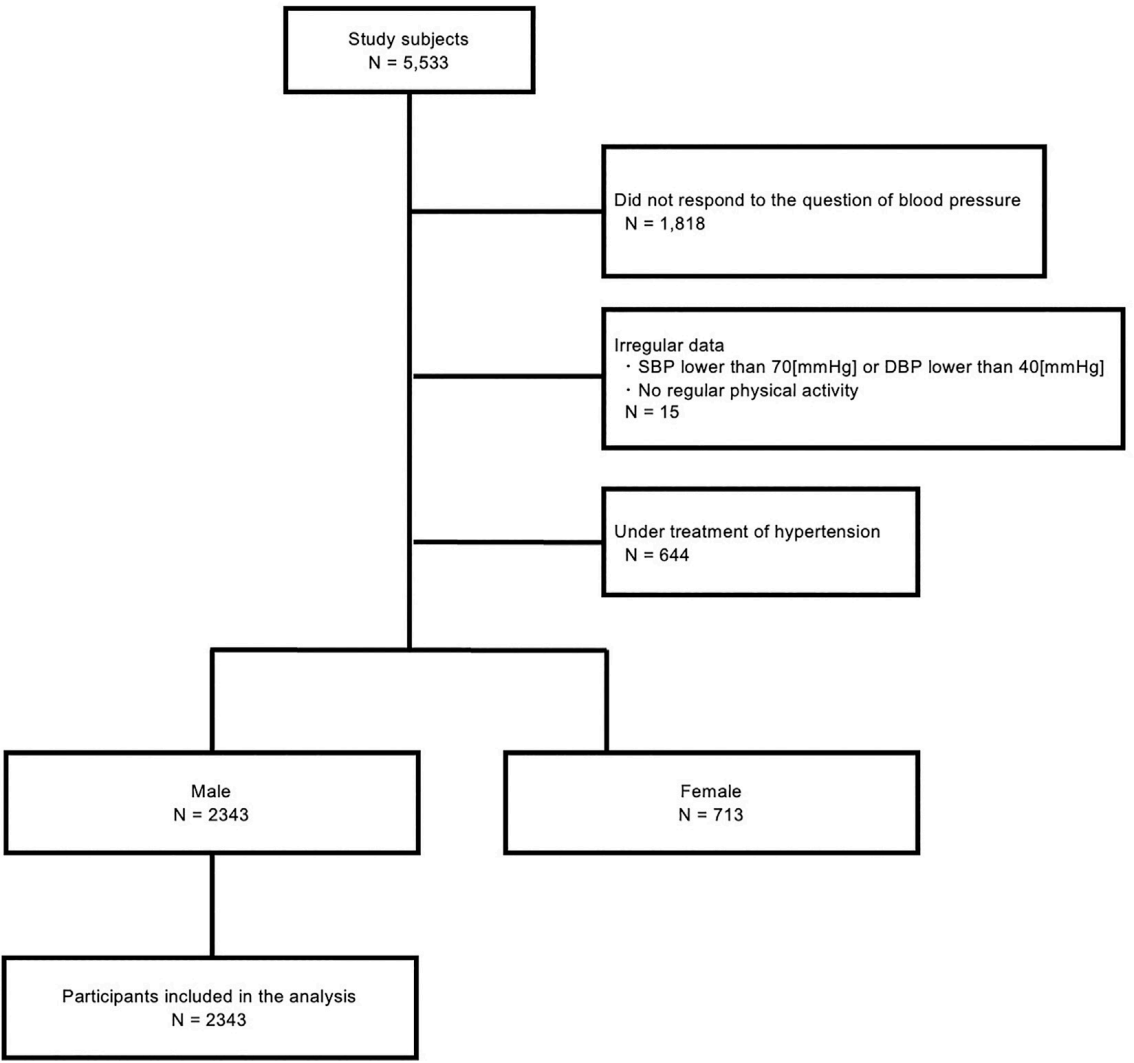
Flow design of the study participants.

We received 5,533 responses and 3,715 of the responses provided their BP. Then, subjects who do not regularly exercise or subjects with SBP lower than 70 mmHg or DBP lower than 40 mmHg, or subjects under treatment of hypertension were excluded (N = 3,056). As the majority of the responses at this point were male (76.7%), the analysis was conducted on male subjects (N = 2,343) (Figure 1).

**FIGURE 2:**
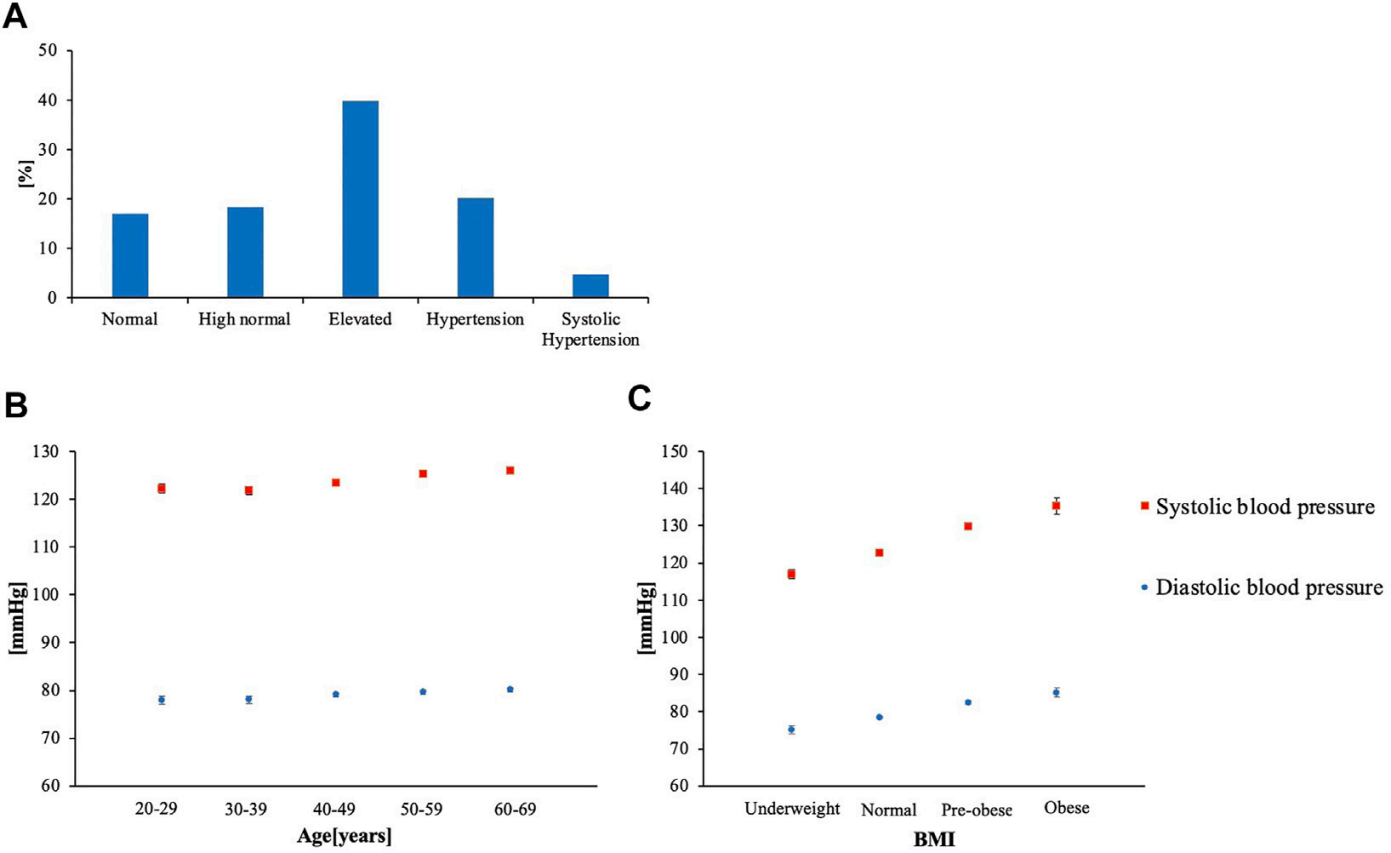
**(A)** Percentage distribution of BP categories **(B)** Age-specific mean BP **(C)** BMI-specific mean BP (Underweight: < 18.5, Normal: 18.5 ≤ BMI < 25.0, pre-obese:25.0 ≤ BMI < 30.0, Obese: 30.0 ≤ BMI).

The age [mean (SE)] was 49.26 (0.236), BMI was 23.20 (0.181), SBP was 124.23 (0.280), and DBP was 79.27 (0.234) (Table 1). Vigorous PA MET-minutes/week was 388.3 (21.29), moderate PA MET-minutes/week was 235.81 (11.57), walking PA MET-minutes/week was 579.56 (18.16), and total PA MET-minutes/week was 1,207.3 (39.06) (Table 1). Over 40% of the subjects had elevated BP (Figure 2A). Both age and BMI showed positive correlation with SBP and DBP (*p* < 0.001) (Figure 2B,C).

**TABLE 1:** Basic characteristics.

	Male (N = 2,343)	
	Mean	SE
Age	49.26	0.236
BMI (kg/m^2^)	23.20	0.181
SBP (mm Hg)	124.2	0.280
DBP (mm Hg)	79.27	0.234
Vigorous PA MET-minutes/week	388.3	21.29
Moderate PA MET-minutes/week	235.8	11.57
Walking MET-minutes/week	579.6	18.16
Total PA MET-minutes/week	1,203.7	39.06

PA: physical activity.

### 3.2 Physical activity frequency, time length, MET-minutes/week, and blood pressure categories

**FIGURE 3:**
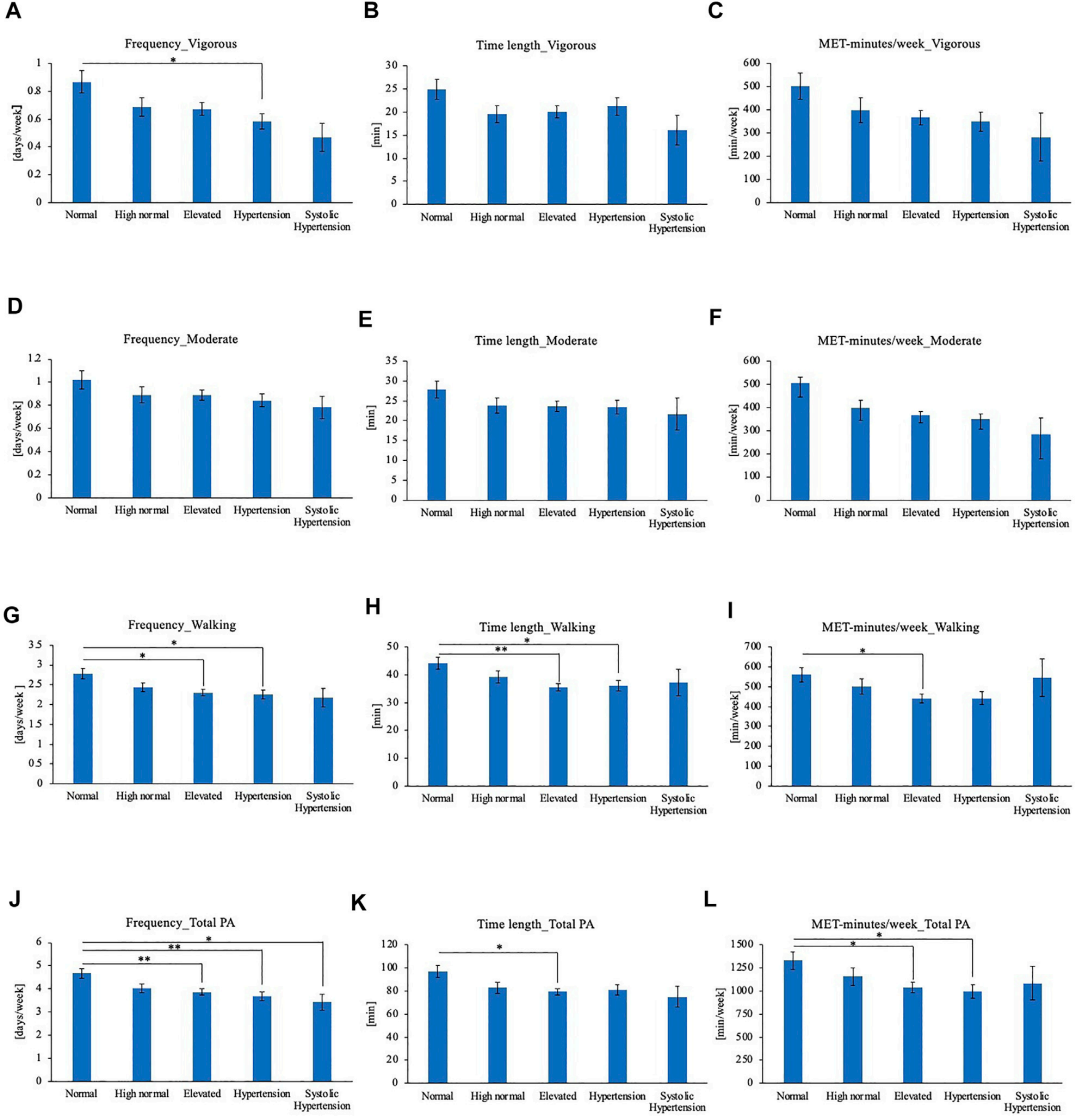
**(A)** Frequency of vigorous physical activity **(B)** Time length of vigorous physical activity **(C)** MET-minutes/week of vigorous physical activity **(D)** Frequency of moderate physical activity **(E)** Time length of moderate physical activity **(F)** MET-minutes/week of moderate physical activity **(G)** Frequency of walking **(H)** Time length of walking **(I)** MET-minutes/week of walking **(J)** Frequency of total physical activity **(K)** Time length of total physical activity **(L)** MET-minutes/week of total physical activity. *p < 0.05, **p < 0.01 (Tukey), PA: Physical activity.

Subjects’ physical activity timings were compared according to their grouping of their BP based on the Japanese blood pressure classification guideline as aforementioned (Umemura et al., 2019). Physical activity frequency, time length, and MET-minutes/week of BP categories were compared for each level of physical activity intensity (Figure 3). For vigorous physical activity, Hypertension group showed lower average frequency compared to Normal BP group (Figures 3A–C). No significant differences were observed for moderate physical activity frequency, time length or MET-minutes/week (Figures 3D–F). For walking, frequency and time length was lower in Elevated BP group and Hypertension group compared to normal BP group (Figures 3G–H). Elevated BP group showed lower walking MET-minutes/week than normal BP group as well (Figure 3I). In comparison of total physical activity, the physical activity frequency of Elevated BP, Hypertension, and Systolic Hypertension group were lower than Normal BP group (Figure 3J). Elevated BP group showed shorter total physical activity time length as well (Figure 3K). Elevated BP group and Hypertension group showed shorter MET-minutes/week for total physical activities (Figure 3L).

### 3.3 Physical activity timing and blood pressure

**FIGURE 4:**
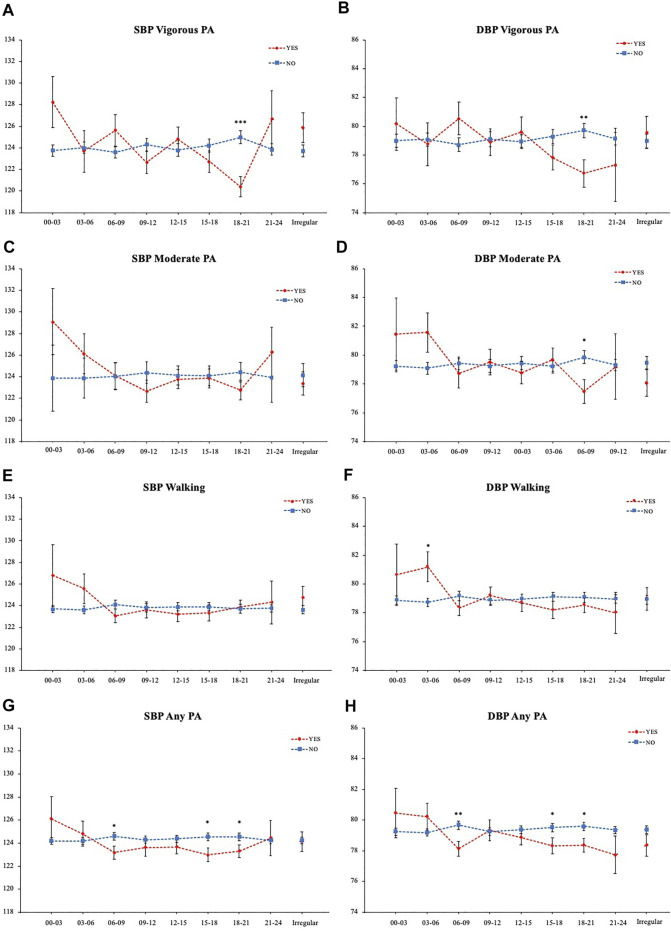
BP comparison of each physical activity timing. **(A)** Vigorous physical activity SBP **(B)** Vigorous physical activity DBP **(C)** Moderate physical activity SBP **(D)** Moderate physical activity DBP **(E)** Walking SBP **(F)** Walking DBP **(G)** Any physical activity SBP **(H)** Any physical activity DBP. *p < 0.05, **p < 0.01, ***p < 0.001 (Student T-test).

Next, to analyze the effect of exercise for each time period, the mean SBP and DBP depending on whether they exercised or not (Yes/No) in each time period were compared individually and plotted in time order (Figure 4). The mean SBP of those who perform vigorous physical activity from 18:00 to 21:00 was 120.40 (0.93), which is significantly lower than those who do not, who had a mean SBP of 124.98 (0.60) with a mean difference of 4.58 mmHg (Figure 4A). The DBP was also 2.99 mmHg lower for those who exercised (Figure 4B). Similarly, those who perform evening moderate physical activity (18:00–21:00) showed significantly lower DBP (Figure 4D). On the other hand, those who walked in the early morning (03:00–06:00) showed 2.47 mmHg higher DBP (Figure 4F). Comparison of those who performed any kind of physical activities in the time period showed that for both SBP and DBP, those who exercised at 06:00–09:00, 15:00–18:00, and 18:00–21:00 had lower average BP (Figures 4G,H).

To investigate the interaction between time periods, multiple regression analysis was conducted. BMI and age were added as confounding factors and the influence between frequency, length, total MET-minutes, and time periods were analyzed. While other time periods or frequency, length, and total MET-minutes did not show associations, vigorous physical activity from 18:00 to 21:00 showed a negative association with both SBP and DBP (Tables 2,3). Moderate physical activity from 18:00–21:00 showed a negative association with DBP, while walking from 03:00–06:00 showed a positive association (Table 3).

**TABLE 2:** Association between physical activity and systolic blood pressure.

	Vigorous physical activity				Moderate physical activity				Walking			
	Β	*p* value	R^2^	F	β	*p* value	R^2^	F	β	*p* value	R^2^	F
Frequency	−0.071	0.238	0.10	7.071	−0.103	0.060	0.038	3.653	−0.053	0.223	0.03	4.484
time length	−0.052	0.367			−0.076	0.159			−0.004	0.939		
total METS	0.021	0.795			0.138	0.056			0.060	0.381		
00:00–03:00	0.058	0.120			0.054	0.101			0.035	0.164		
03:00–06:00	0.015	0.695			0.034	0.322			0.044	0.091		
06:00–09:00	0.075	0.050			0.004	0.905			−0.016	0.571		
09:00–12:00	−0.048	0.216			−0.053	0.132			0.006	0.809		
12:00–15:00	0.060	0.118			−0.010	0.784			−0.021	0.432		
15:00–18:00	−0.039	0.288			−0.005	0.875			−0.001	0.963		
18:00–21:00	−0.086	0.034*			−0.068	0.062			0.026	0.348		
21:00–24:00	0.047	0.190			0.034	0.298			0.016	0.523		
Irregular	0.025	0.549			−0.035	0.362			0.033	0.265		

* *p* < 0.05; Multivariable regression analyses adjusted by age and BMI.

**TABLE 3:** Association between physical activity and diastolic blood pressure.

	Vigorous physical activity				Moderate physical activity				Walking			
	Β	*p* value	R^2^	F	β	*p* value	R^2^	F	β	*p* value	R^2^	F
Frequency	0.019	0.761	0.033	2.88	0.009	0.868	0.014	1.972	−0.026	0.562	0.012	2.402
time length	0.048	0.421			−0.023	0.670			−0.050	0.358		
total METS	−0.059	0.469			−0.013	0.861			0.047	0.496		
00:00–03:00	0.001	0.982			0.016	0.622			0.019	0.450		
03:00–06:00	−0.011	0.777			0.037	0.286			0.055	0.036*		
06:00–09:00	0.040	0.316			−0.033	0.333			−0.022	0.438		
09:00–12:00	−0.040	0.324			−0.002	0.962			0.022	0.413		
12:00–15:00	0.013	0.733			−0.045	0.212			−0.012	0.657		
15:00–18:00	−0.065	0.088			0.006	0.850			−0.032	0.219		
18:00–21:00	−0.094	0.025*			−0.106	0.004**			−0.017	0.545		
21:00–24:00	−0.038	0.307			−0.005	0.881			−0.015	0.543		
Irregular	−0.028	0.522			−0.070	0.069			−0.006	0.841		

* *p* < 0.05; **, *p* < 0.01 Multivariable regression analyses adjusted by age and BMI.

### 3.4 Blood pressure categories and physical activity timings

Subjects’ physical activity timings were compared according to their grouping of their BP based on the Japanese blood pressure classification guideline as aforementioned (Umemura et al., 2019).

**FIGURE 5:**
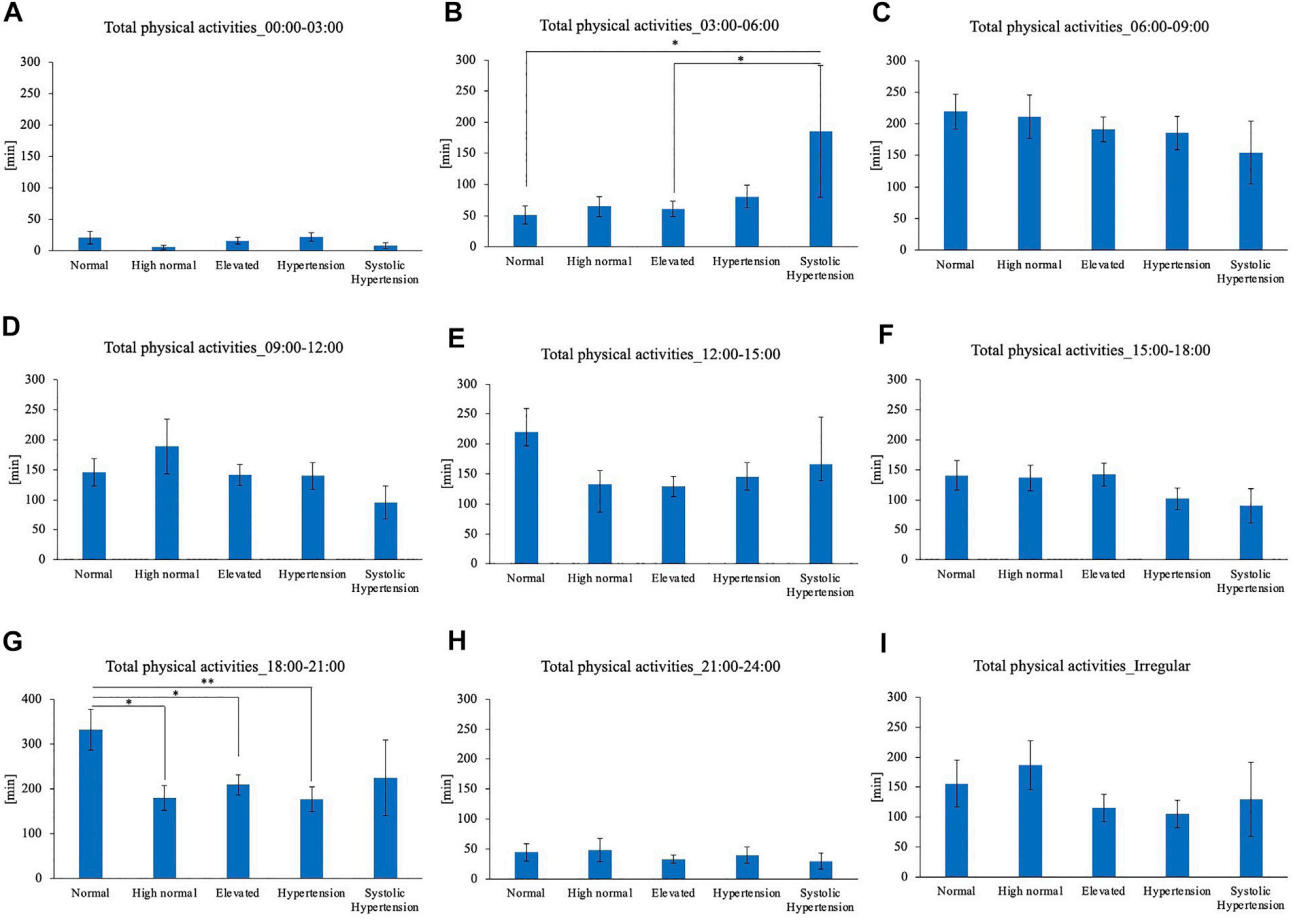
BP categories and total physical activities for each time period **(A)** 00:00–03:00 **(B)** 03:00–06:00 **(C)** 06:00–09:00 **(D)** 09:00–12:00 **(E)** 12:00–15:00 **(F)** 15:00–18:00 **(G)** 18:00–21:00 **(H)** 21:00–24:00 **(I)** Irregular timing*p < 0.05, **p < 0.01 (Tukey).

**FIGURE 6:**
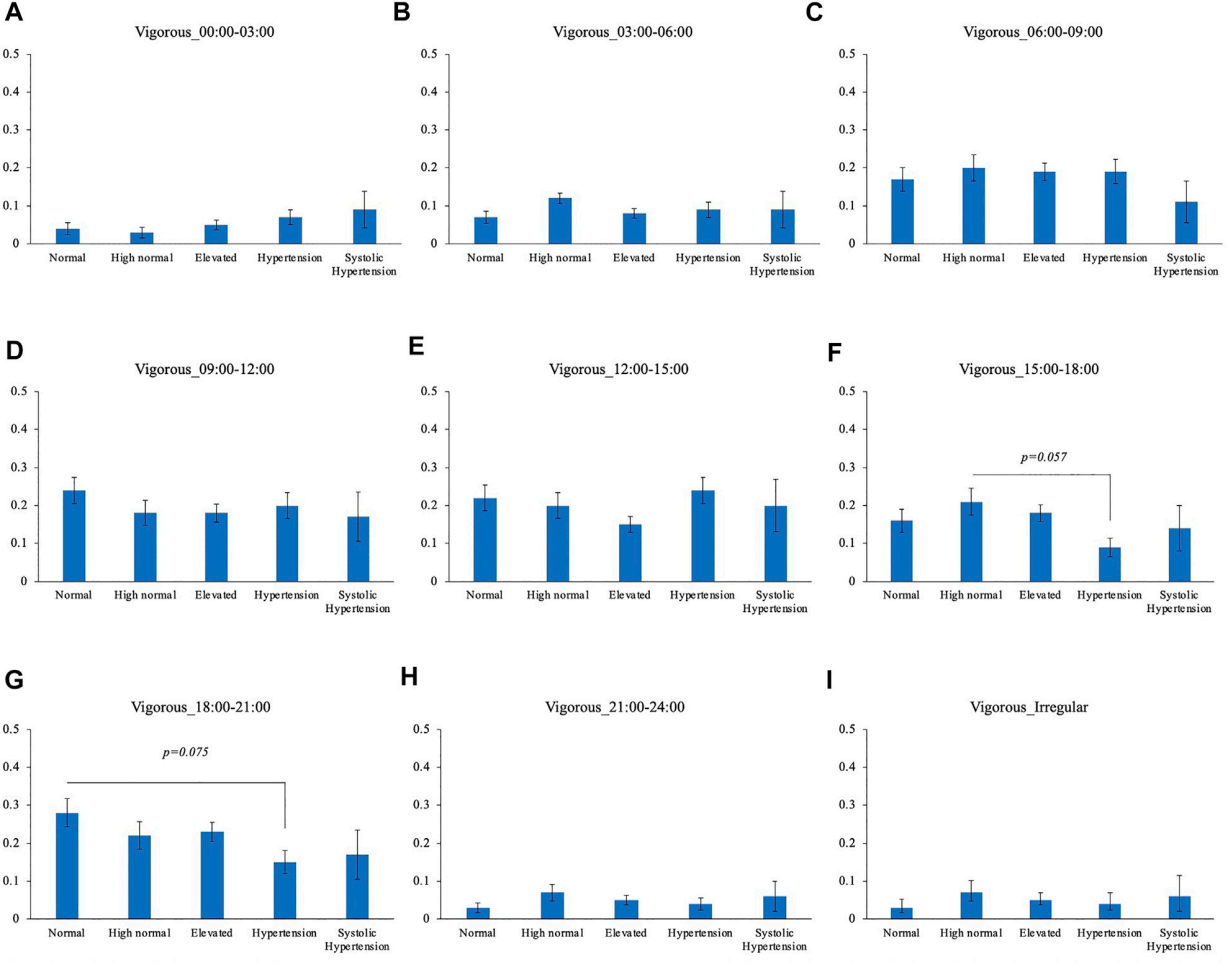
BP categories and vigorous physical activity frequency for each time period **(A)** 00:00–03:00 **(B)** 03:00–06:00 **(C)** 06:00–09:00 **(D)** 09:00–12:00 **(E)** 12:00–15:00 **(F)** 15:00–18:00 **(G)** 18:00–21:00 **(H)** 21:00–24:00 **(I)** Irregular timing*p < 0.05 (Tukey).

**FIGURE 7:**
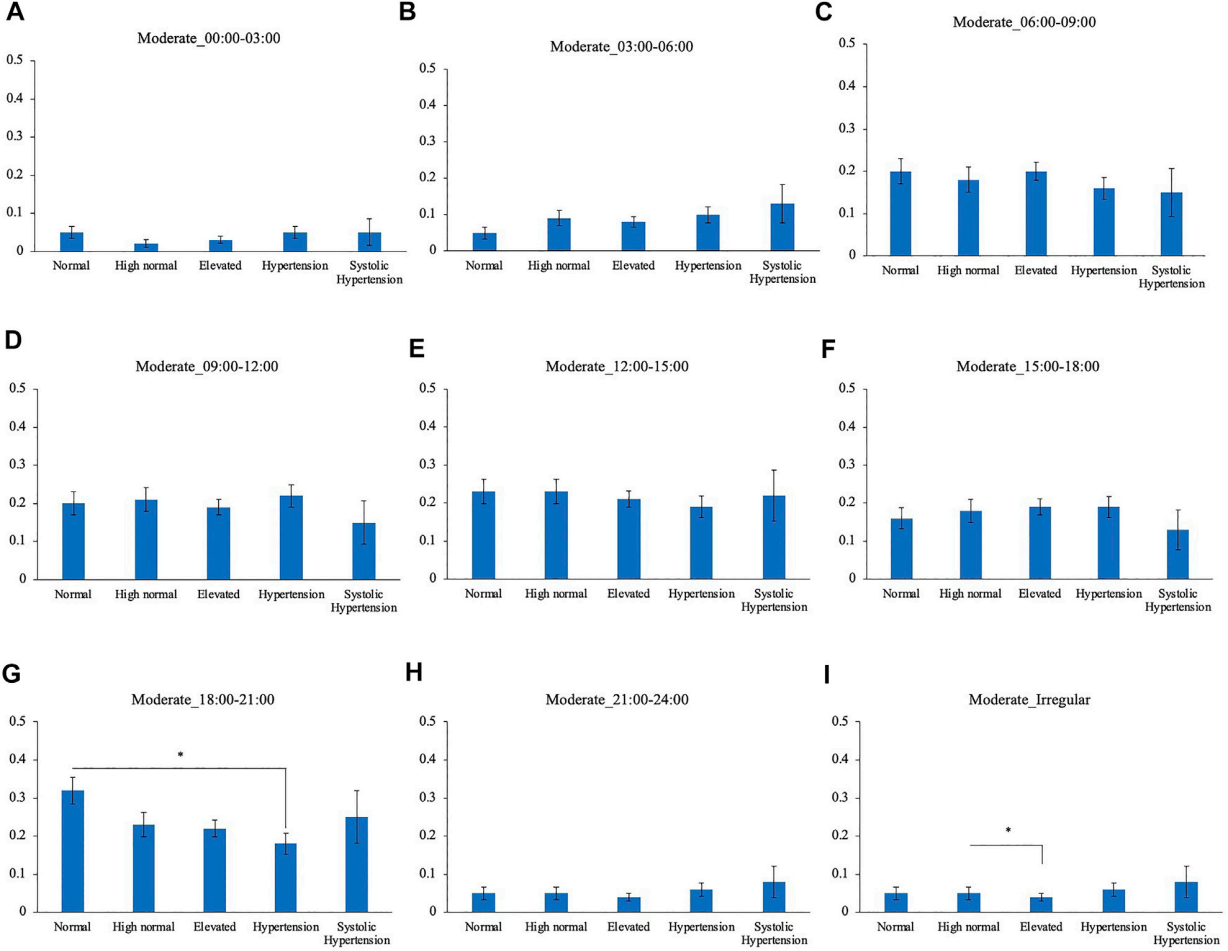
BP categories and moderate physical activity frequency for each time period **(A)** 00:00–03:00 **(B)** 03:00–06:00 **(C)** 06:00–09:00 **(D)** 09:00–12:00 **(E)** 12:00–15:00 **(F)** 15:00–18:00 **(G)** 18:00–21:00 **(H)** 21:00–24:00 **(I)** Irregular timing *p < 0.05 (Tukey).

**FIGURE 8:**
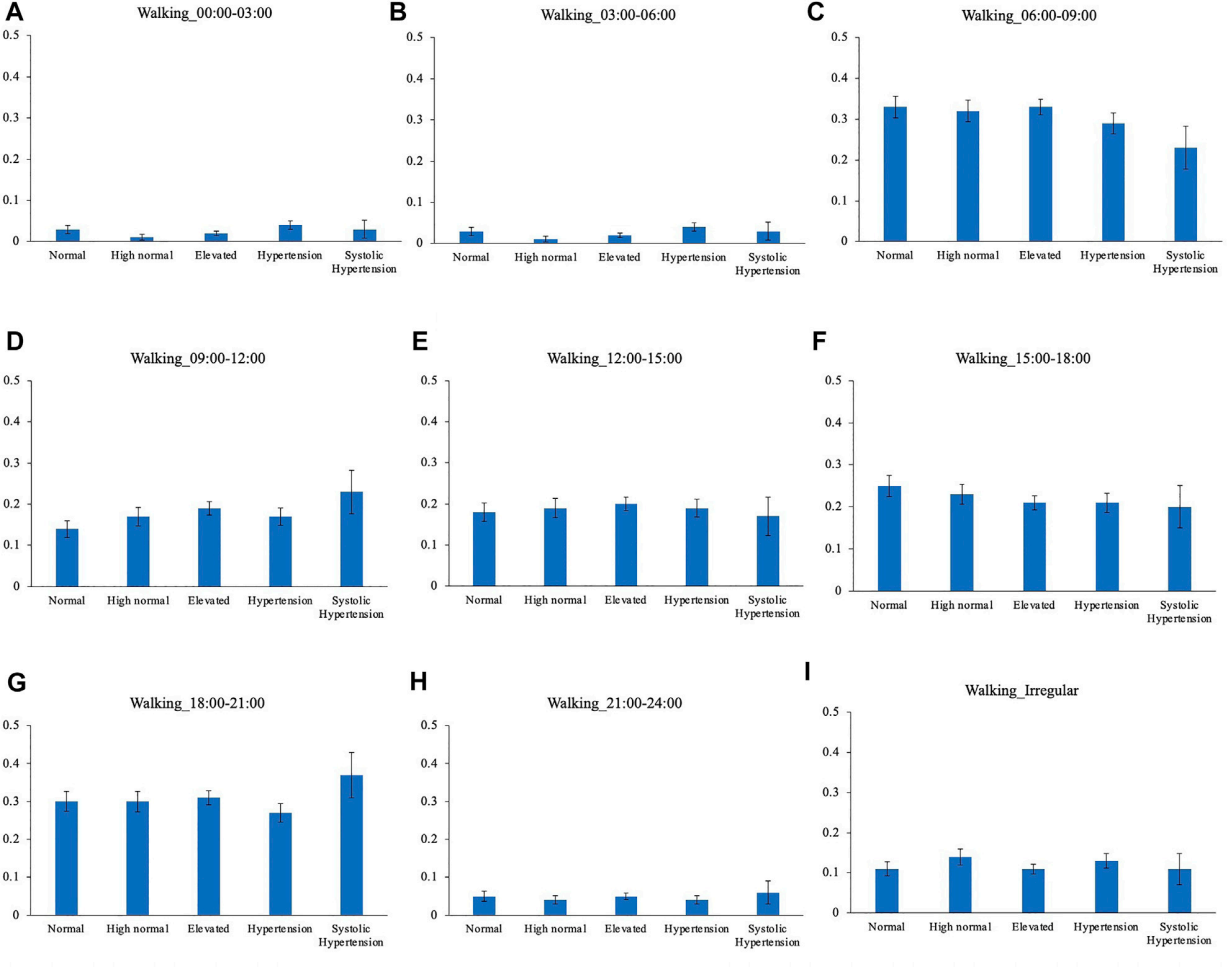
BP categories and walking frequency for each time period. **(A)** 00:00–03:00 **(B)** 03:00–06:00 **(C)** 06:00–09:00 **(D)** 09:00–12:00 **(E)** 12:00–15:00 **(F)** 15:00–18:00 **(G)** 18:00–21:00 **(H)** 21:00–24:00 **(I)** Irregular timing.

Comparison of the timing of total physical activity time showed that Systolic hypertension group had an average longer physical activity from 03:00 to 06:00. Normal BP group exercised longer from 18:00 to 21:00 compared to High normal BP group, Elevated BP group and Hypertension group (Figure 5). Focusing exclusively on the timing of the physical activity for each intensity level, Normal BP group tended to perform moderate physical activity more from 18:00 to 21:00 (Figure 6). The same tendency could be observed with vigorous physical activity (Figure 7). Walking did not show a significant difference among groups (Figure 8).

## 4 Discussion

In this study, we investigated the association between BP and the timing of physical activities for different levels of intensity. Normal BP groups had higher frequency, time length, and MET-minutes/week of total physical activity compared to Elevated BP group, suggesting that the overall effect of exercise on BP is beneficial, consistent with previous findings. However, our results indicated that time-specific associations exist between BP and each intensity level of physical activity. From the comparison of SBP and DBP between those who performed physical activity at each time period, vigorous or moderate physical activity in the evening (18:00–21:00) showed significantly lower average BP. On the other hand, those who walked in the early morning (03:00–06:00) showed higher DBP. These time-specific differences were confirmed from multiple regression analysis. While other time periods or frequency, length, and total MET-minutes did not show associations, vigorous physical activity from 18:00 to 21:00 showed a negative association with both SBP and DBP. Moderate physical activity from 18:00–21:00 showed a negative association with DBP, while walking from 03:00–06:00 showed a positive association. Furthermore, normotensive subjects performed vigorous or moderate evening (18:00 to 21:00) physical activity more often compared to pre-hypertensive or hypertensive subjects and showed significantly lower SBP and DBP. SBP showed the largest difference of 4.58 mmHg.

Our research subjects included both normotensive subjects and hypertensive subjects. Although the effect of exercise is different depending on the subjects’ characteristics and exercise type, our findings resemble other reported magnitudes of BP reduction. When BP changes resulting from exercise were compared in a meta-analysis, endurance training reduced 3.5 mmHg [confidence limits −4.6 to −2.3] SBP and 2.5 mmHg [−3.2 to −1.7] DBP (Cornelissen and Smart, 2013). Furthermore, it has been reported that in the middle-aged, 2 mmHg lower SBP is related to about 10% lower stroke mortality and about 7% lower mortality from cardiovascular causes (Lewington et al., 2002). Another meta-analysis reported that in a biracial population-wide cohort, 1 mmHg decrement of SBP could prevent a substantial number of cardiovascular events, especially heart failure (Hardy et al., 2015). Therefore, considering the magnitude of BP difference, the effect of evening vigorous exercise is suggested to be clinically beneficial.

The time-of-day exercise effects on BP have been discussed in various papers. Jones et al. have reported that a single bout of aerobic exercise, categorized in moderate or vigorous physical activity performed in the evening, reduces the mean arterial BP while the morning exercise led to a slight increase (Jones et al., 2008). This diurnal variation in the PEH has been supported with evidence from further research conducted under continuous and intermittent exercise protocols (Jones et al., 2009). However, it has been shown that the morning hypotensive effect may be masked by the morning surge, the circadian rising of the BP in the morning. When the SBP was compared within morning trials (control versus exercise) and evening trials to take the circadian variation into account, a greater decrease occurred in the morning (de Brito et al., 2015). On the other hand, in treated hypertensive men, it has been reported that evening aerobic exercise for 10 weeks decreased clinic and ambulatory BP while morning trials did not (Brito et al., 2019). Another study conducted morning aerobic exercise for 2 months on healthy young subjects, and no significant changes in BP were observed (Shiotani et al., 2009). Therefore, unlike the acute hypotensive effects of morning exercise, the morning chronic hypotensive effects have not been observed. Taken together with our results, it is suggested that morning exercise may show greater acute PEH considering the circadian variation, but in the long term, for the aim of preventing hypertension, evening exercise may be more effective.

Those who walked in the early morning (03:00 to 06:00) had higher mean DBP than those who did not. Hours at the end of the sleep period and before awakening are known to have the highest incidence of major cardiovascular events (Wolk et al., 2005). Acute early morning exercise immediately after awakening from nocturnal sleep has been reported to be associated with a post-exercise rise in mean BP (Jones et al., 2008). Also, cold exposure during sleep has been reported to elevate the amplitude of morning BP surge (Kuo et al., 2014). Morning BP surge is closely related with the 24-h BP profile (Bilo et al., 2018). Walking outside in the early morning may have resulted in cold exposure, leading to an elevated BP.

While walking showed no differences in mean BP for other time periods, BP category comparison showed that Normal BP groups walk more than elevated BP groups, suggesting that consistent with previous reports, walking is beneficial for high BP prevention. Walking at timings other than early mornings may be better to avoid a detrimental effect on BP.

One limitation of our study is that this is an analysis of a questionnaire data answered by individuals, and it may include some inaccuracy. An intervention study is needed to confirm the findings. Second, because the reported BP was from a medical checkup/examination, a condition which BP in office is elevated while ambulatory or home BP is normal, known as white-coat hypertension, may have occurred. There is general agreement that white-coat hypertension has a greater risk than normotension, but for more accurate analysis, further consideration is needed (Mancia et al., 2021). Third, we only categorized the physical activity with vigorous physical activity, moderate physical activity, and walking, and could not classify whether the type of physical activity was aerobic exercise or resistance training. Analyzing according to the exercise type would provide a clearer view. Another limitation of the analysis was that we do not know whether the subjects were dippers or non-dippers. It has been reported that the timing-dependent blood lowering effect of exercise is different in dipping and non-dipping subjects (Park et al., 2005).

## 5 Conclusion

In conclusion, among normotensive and hypertensive, unmedicated Japanese men, normotensive subjects performed vigorous or moderate evening physical activity more often. Furthermore, the average BP of subjects who performed vigorous or moderate evening physical activity was lower, while walking in the early morning suggested a detrimental association.

## Data Availability

The data are not publicly available because of patent preparation. Data will be sent upon request from the corresponding author.
